# Psychophysiological Characteristics of Burnout Syndrome: Resting-State EEG Analysis

**DOI:** 10.1155/2019/3764354

**Published:** 2019-07-29

**Authors:** Krystyna Golonka, Magda Gawlowska, Justyna Mojsa-Kaja, Tadeusz Marek

**Affiliations:** Institute of Applied Psychology, Faculty of Management and Social Communication, Jagiellonian University, Łojasiewicza 4, 30-348 Kraków, Poland

## Abstract

**Introduction:**

The consequences of chronic work-related stress are related to various emotional, cognitive, and behavioral symptoms. Occupational burnout as a complex syndrome is characterized by exhaustion, cynicism, and lower professional efficacy. Moreover, the growing amount of research on the neural correlates of burnout broadens the existing knowledge on the mechanisms underlying this syndrome.

**Aim of the Study:**

The aim of the study is to explore possible differences in brain activity between burnout and nonburnout employees. Frequency-specific EEG power analyses in a resting-state condition in burnout subjects and controls are presented.

**Materials and Methods:**

Burnout employees (N=46; 19 men) were matched with the control group (N=49; 19 men; mean age: 36.14 years, SD=7.89). The Maslach Burnout Inventory–General Survey (MBI-GS) and the Areas of Worklife Survey (AWS) scale were used to measure burnout symptoms and work conditions, respectively. A 256-channel EEG (EGI System 300) was used to collect psychophysiological data. A repeated measures ANOVA was performed with condition (eyes-open vs. eyes-closed) and region (6 levels: extracted scalp regions) factors; burnout (2 levels: burnout vs. no burnout) was the grouping factor.

**Results:**

A significant difference was observed only in the alpha frequency band: the burnout group revealed significantly lower alpha power in the eyes-open condition compared to the controls (p<0.05). The correlation analysis revealed that gender may significantly change the pattern of relations between EEG spectral characteristics and burnout symptoms.

**Conclusions:**

Reduced alpha power in burnout individuals suggests cortical hyperactivity and may be related to greater mental effort and the possible development of compensatory mechanisms by burnout subjects.

## 1. Introduction

Burnout syndrome is defined as a process of psychological reaction to long-term work-related stress [1] which is influenced by individual and contextual factors [2]. According to the latest 11^th^ International Classification of Diseases (ICD-11), burnout is included among “Factors influencing health status or contact with health services” in the section “Problems associated with employment or unemployment” (code: QD85) and refers to workplace stress that has not been effectively managed [3]. In ICD-11, burnout is conceptualized as an occupational phenomenon that is specifically related to experiences in the professional context and is not classified as a medical condition. World Health Organization characterizes burnout by three dimensions: “(1) feelings of energy depletion or exhaustion; (2) increased mental distance from one's job, or feelings of negativism or cynicism related to one's job; and (3) reduced professional efficacy” [3]. It directly corresponds to Maslach, Jackson, & Leiter [4] who described burnout as a state of exhaustion, depersonalization or cynicism, and low professional efficacy. Some researchers emphasize however that the main components of burnout syndrome are psychophysical exhaustion and psychological distancing from work [5].

Burnout research has significantly developed in recent years and expanded over various research areas. The first studies on burnout were related to work and organizational psychology [1, 6–8], but further research on burnout syndrome is also relevant to clinical psychology [9–14], neuropsychology [15, 16], neurophysiology [17–19], and neuroscience [20–27]. It seems that burnout syndrome has become a popular research area for three reasons: (1) its prevalence in the general population of employees; (2) significant individual and organizational consequences; and (3) important scientific dispute on its etiology and the symptomatic characteristics that differentiate it from other diseases, especially from depression [9, 28]. Regarding methodology in burnout studies, objective methods and research outcomes are particularly needed to answer the question of whether severe burnout syndrome may be a separate entity, or whether it is a form of depression or anxiety-depression disorder induced by long-term work-related stress.

Neuroimaging research revealed that burnout or prolonged occupational stress correlated with specific anatomical and functional brain characteristics [22, 23, 25, 26]. For example, Jovanovic et al. [23] showed that subjects with chronic work-related stress revealed functional disconnection between the amygdala and the medial prefrontal cortex (mPFC), including anterior cingulate cortex (ACC). Moreover, they observed that receptors which are involved in the HPA regulation (5-HT1A receptors) were reduced in the ACC, the insular cortex, and in the hippocampus. These results indicate significant structural and functional brain changes and may suggest impaired top-down regulation of stress in subjects with prolonged work-related stress [23]. Similarly, Blix, Perski, Berglund, & Savic [26] analyzing the sample with chronic occupational stress observed reduction in the grey matter volumes of the ACC and the dorsolateral prefrontal cortex (dPFC), and reduced volumes of caudate and putamen. Savic [25] observed that burnout patients demonstrated significantly thinner mesial frontal cortex and selective changes in subcortical volumes: their amygdala volumes were bilaterally increased and caudate volumes were decreased. Golkar et al. [22] observed weaker activation of the functional network between the right amygdala and the anterior cingulate cortex in burnout subjects what may explain difficulties in controlling and coping with negative emotions. These studies give a solid basis for further exploration of neural correlates of burnout and search for its neurophysiological indicators.

In previous psychophysiological studies using electroencephalography (EEG), cognitive impairments in burnout subjects, accompanied by a changed pattern of selected Event-Related Potentials (ERP), were observed [29–33]. In our earlier study, we observed altered ERP pattern of processing of emotion-related stimuli in burnout subjects, which may explain one of the core burnout components: depersonalization/cynicism [15]. Additionally, Luijtelaar and colleagues [29] analyzed frequency-specific EEG power and revealed that lower alpha peak frequency and reduced beta power were observed in burnout subjects. Frequency-specific EEG power analyses may be an interesting perspective in exploring burnout and may bring additional insights in the characteristics of burnout syndrome. These explorations in relation to burnout may be particularly interesting in terms of such burnout characteristics as mental fatigue, depletion of energy, and a state of exhaustion [1, 7, 34–38]. Some studies clearly showed that burnout subjects demonstrate specific arousal patterns such as lower energy levels and higher levels of tension [39, 40]. In this context, the indexes of arousal levels and reactivity may be of particular interest. According to Fonseca, Tedrus, Bianchini, & Silva [41], in resting conditions, the differences in alpha EEG activity between eyes-closed and eyes-open conditions could be used as a measure of resting-state arousal. Arousal level may refer to a reduction in absolute power in the eyes-open condition (EO) as compared to the eyes-closed condition (EC). Another index of arousal, the level of reactivity that may be assessed by alpha reactivity index, counted as a quotient of absolute alpha power in EO to absolute alpha power in EC (the greater alpha reactivity index relates to lower reactivity) [41].

Regarding the overlapping effects with depression [10, 28–30, 42], it is particularly interesting to analyze frontal alpha asymmetry (FAA) in burnout. In depression, frontal alpha (8–13 Hz) asymmetry with hypoactivity in the frontal lobe has been reported in many findings [43–45], so FAA may also be observed in burnout groups. However, some studies indicate that the greater right alpha activity in depression relates to small to medium effect sizes [46] and that this tendency is not evident [47]. One of the latest meta-analysis on FAA in depression [48] confirms these ambiguities, indicating the limited diagnostic value of FAA in major depressive disorders. Moreover, a previous study on EEG spectral analysis in a burnout group did not reveal FAA [29]. In the light of these findings, it is difficult to conclude whether the alpha asymmetry is typical of burnout subjects.

In this study, we aim to analyze the spectral characteristics of resting-state EEG and compare them between burnout subjects and controls. Referring to a previous study on spectral power analysis in burnout [29], we expect to find significant differences between burnout subjects and controls in *α* (8.5–13.0 Hz) and *β* (13.5–30 Hz) frequency. In comparison to the control group, our hypotheses are as follows: (H1) significantly lower alpha peak frequency will be observed in the burnout group; (H2) significantly lower beta power will be observed in the burnout group; (H3) the burnout group will not be differentiated by frontal alpha asymmetry. Referring to van Luijtelaar et al.'s study [29], we will compare resting EEG in the eyes-open and eyes-closed conditions. Furthermore, with reference to Tement et al.'s [49] study, we expect to observe differences in alpha power in resting EEG; however, no specific hypotheses were formulated due to the differences in the study sample and methodology (students; only eyes-closed condition; regression models).

## 2. Materials and Methods

### 2.1. Participants

Subjects were recruited from 272 volunteers who responded to an invitation describing the project's aim and a short description of the study. The invitation was presented on business social networks and sent in emails to public and private organizations. The inclusion criteria for the study were as follows: employee status (active workers with higher education and at least 1.5 years of work experience, working in a day-shift system), right-handedness, correct or corrected-to-normal vision, addiction free, no history of neurological or psychiatric diseases, and not pregnant. The initial sample consisted of 100 participants (40 men). Due to poor spectral EEG data quality and ambiguous burnout characteristics, 5 participants were excluded. The study sample (N=95) consisted of the burnout group (N=46; 19 men), which was matched with the control group (N=49; 19 men) in terms of gender and age characteristics (mean age: 36.14 years, SD=7.89).

The study protocol was approved by the Bioethics Commission of Jagiellonian University and was carried out in accordance with the recommendations of the APA Ethics Code. Participants were paid for their contribution in the project. Each subject gave written informed consent.

The burnout group consisted of participants who had high scores on burnout measure and who reported their job-related context as stressful. Burnout and job context were assessed using Polish versions of the Maslach Burnout Inventory–General Survey (MBI-GS) [3] and the Areas of Worklife Survey scale (AWS) [50].

The MBI-GS consists of 16 items rated on a 7-point scale ranging from 0 “never” to 6 “every day.” The instrument measures three dimensions of burnout: exhaustion (5 items), cynicism (5 items), and professional efficacy (6 items). Cronbach's *α* coefficients based on the sample are *α*_exhaustion_ = 0.922, *α*_cynicism_ = 0.9101, and *α*_efficacy_ = 0.889.

The AWS consists of 29 items which relate to work conditions and assess employees' perceived alignment between their work environment and individual preferences. Six areas of worklife are analyzed: workload (6 items), control (3 items), reward (4 items), community (5 items), fairness (6 items), and values (5 items). They are rated on a 5-point scale ranging from 1 “strongly disagree” to 5 ”strongly agree.” Cronbach's *α* coefficients were *α*_workload_ = 0.848, *α*_control_ = 0.803, *α*_reward_ = 0.839, *α*_community_ = 0.894, *α*_fairness_ = 0.864, and *α*_values_ = 0.757.

The burnout group comprises participants who scored high (>3) on the two burnout dimensions of exhaustion and cynicism, and low scores (< 3) in at least three AWS scales; this indicated higher burnout symptoms and more stressful work-related context, as assessed by a lower degree of matching between the individual's workplace and preferences.

### 2.2. Experimental Procedure

The EEG data was recorded for 3 minutes for the eyes-open and 3 minutes for the eyes-closed condition. Subjects were asked to sit still and focus on the fixation point; when their eyes were closed, they were asked to sit still with closed eyes.

### 2.3. EEG Analysis

Continuous dense-array EEG data (HydroCel Geodesic Sensor Net, EGI System 300; Electrical Geodesic Inc., OR, USA) was collected from a 256-channel EEG at a sampling rate of 250 Hz (band-pass filtered at 0.01–100 Hz with a vertex electrode as a reference) and recorded with NetStation Software (Version 4.5.1, Electrical Geodesic Inc., OR, USA). The impedance for all electrodes was kept below 50 kΩ. The offline data analysis was conducted with the open-source EEGLAB toolbox [51]. Before the preprocessing steps, facial electrodes were removed; thus, further analysis was performed on 224 channels. Data was digitally filtered to remove frequencies below 0.5 Hz and above 35 Hz. Average reference was recomputed, and bad channels were automatically removed by kurtosis measures with a threshold value of 5 standard deviations. Next, continuous data was visually inspected in order to manually remove channels or time epochs containing high-amplitude, high-frequency muscle noise, and other irregular artifacts.

Independent component analysis was used to remove artifacts from data. Due to the large number of channels, decomposition of EEG data with the Infomax algorithm was preceded with Principle Component Analysis. Fifty independent components were extracted and visually inspected for each subject. On the basis of the spatiotemporal pattern [52, 53], components recognized as blinks, heart rate, saccades, muscle artifacts, or bad channels were removed. Missing channels were interpolated, and ICA weights were recomputed. Data was divided into the eyes-open (EO) and eyes-closed (EC) conditions. Spectral decomposition was performed using the Welch window, followed by Fast Fourier Transform (FFT). Mean power spectra for alpha (8–13 Hz), beta (14–35 Hz), delta (1–3 Hz), and theta (4–7 Hz.) were extracted for every participant from the electrode clusters localized at the left and right anterior, left and right central, and left and right posterior scalp sites.

## 3. Results and Discussion

The statistical analyses were performed for each frequency band separately. There was no significant effect between the groups for the beta, delta, and theta bands; thus, the statistical analyses will be presented only for the alpha frequency band.

**Figure 1:**
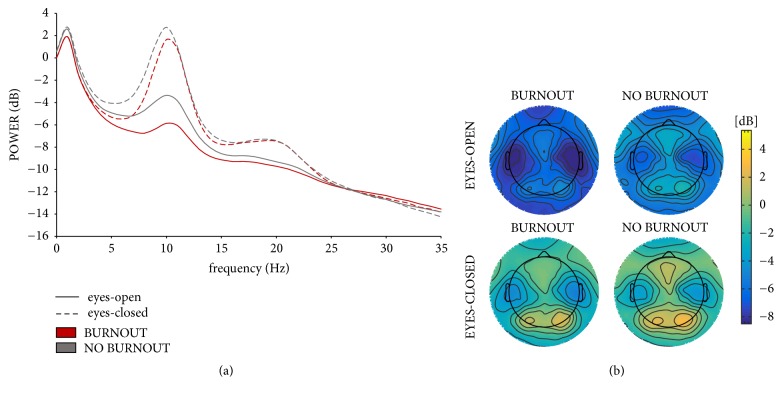
(a) Power spectra in the eyes-open and eyes-closed conditions for the burnout and no burnout groups. (b) Alpha power topography in the eyes-open and eyes-closed condition for the burnout and no burnout groups.

The repeated measures ANOVA was performed with condition (EO vs. EC) and region (6 levels: extracted scalp regions) factors; burnout (2 levels: burnout vs. no burnout) was the grouping factor. As expected, there was a main effect of condition (F_(1,93)_=341.82, p < .001, *η*_*p*_^2^ = 0.786), and alpha power was significantly higher for closed eyes. Moreover, an interaction effect of group and condition was observed (F_(1,93)_=5.43, *p* < .05, *η*_*p*_^2^ = 0.055). The post hoc analysis revealed that there was a significant difference in the OE condition (*p*<.05), with a lower alpha power for the burnout vs. no burnout group (see Figure 1). Finally, there was a significant main effect of scalp region (F_(5,465)_=82.04, *p* < .001, *η*_*p*_^2^ = 0.469) and an interaction effect of condition and scalp region (F_(5,465)_=52.51, *p* < .001, *η*_*p*_^2^ = 0.361). However, these effects were not modulated by burnout occurrence; thus, we neither explored nor interpreted these effects. No significant differences were observed in alpha individual peak frequency between the studied groups.

Thus, we observed significantly lower alpha power in the burnout group in the eyes-open condition. Our results do not support hypothesis 1, which relates to lower alpha peak frequency, or hypothesis 2, which relates to lower beta power in burnout subjects. No significant group or interaction effect was observed. Our results support hypothesis 3, i.e., frontal alpha asymmetry is not observed in burnout subjects. This is in line with Luijtelaar et al.'s [29] observations.

Although to the best of the authors' knowledge higher alpha power has not been observed in any burnout group, this tendency could be expected as burnout reveals some symptomatological similarities to fatigue and depression, for which elevated alpha power has been reported [29, 49]. Thus, the presented results show a novel characteristic in burnout subjects, indicating cortical hyperactivity rather than hypoactivity, which is typical of depression and fatigue.

In further correlation analysis, in the eyes-open (Table 1) and eyes-closed (Table 2) conditions, we observed a significant relation between alpha power and two burnout symptoms: exhaustion and cynicism. For exhaustion, a significant negative correlation was revealed in the eyes-open condition for the anterior, central, and posterior areas. This was observed as global effect for each region and for all left and right areas. In the eyes-closed condition, a significant correlation was observed only for the anterior (globally and hemispheric), central left, and posterior global and left areas. In the eyes-closed condition, the correlation coefficients were weaker compared to the eyes-open condition. For cynicism, significant negative correlations were observed in the eyes-open condition for the global anterior and posterior areas and for both the left and right sides. Weaker correlations were observed for the central global and central left regions.

Further alpha power analysis took gender into account as an important characteristic which may reverse the pattern of relations between alpha power and burnout symptoms [49]. In line with Tement et al.'s findings [49], we observed that alpha power significantly correlated with burnout only in the male subjects (N=38). In females (N=57), although the tendency for negative correlation remains, the relations between alpha power and burnout symptoms in most areas failed to reach significance. The only significant negative correlation between exhaustion and alpha power was observed in the anterior right in the eyes-open condition (r= 0.32, p=0.049). In male subjects, a significant negative correlation was observed between alpha power and cynicism for all areas in the eyes-open condition. These relations were observed for global analyses for the anterior (r= -0.37, p=0.021), central (r= -0.37, p=0.023), and posterior (r= -0.35, p=0.032) areas, as well for hemispheric analyses (anterior left: r= -0.41, p=0.011; right: r= -0.34, p=0.036; central left: r= -0.37, p=0.021; right: r= -0.35, p=0.029; posterior left: r= -0.35, p=0.033; right: r= -0.35, p=0.033). Interestingly, for male subjects, additional significant correlations were found between alpha power and efficacy; all these correlations were positive and were noticed only in the eyes-open condition (anterior left: r= 0.33, p=0.042; central global: r= 0.39, p=0.016, left: r= 0.42, p=0.009, and right: r= 0.36, p=0.028; posterior global: r= 0.32, p=0.048, and left: r= 0.34, p=0.035). These analyses reveal that gender may significantly change the pattern of relations between spectral EEG characteristics and burnout symptoms, thus supporting the findings and conclusions of Tement et al. [49].

Further analysis is based on the index of alpha power in the eyes-open condition, referenced to the eyes-closed resting condition, which is defined as the task-related power decrease/increase (TRPD/TRPI). This index is calculated as TRPD/TRPI% = (EO-EC)/EC x 100 [54–56] and is described as a valuable measure of cortical reactivity. A task-related power decrease (TRPD) of EEG alpha rhythms at about 8–12 Hz reflects cortical activation, while a task-related power increase reflects cortical deactivation [54]. Our analyses on the TRPD index revealed significant differences between the study groups in the central right (F_(1,93)_=6.78,* p*<.05, *η*_*p*_^2^=0,068) and the posterior area (F_(1,93)_=5.86,* p*<.05, *η*_*p*_^2^ = 0,059), indicating a higher TRPD index in burnout subjects. We also noticed a significant positive correlation between TRPD in the central right region and cynicism (r= 0.27, p=0.009). This may suggest that burnout correlates with the TRPD index, showing that greater cynicism is related to a higher TRPD index, which reflects lower cortical activation in the right central brain areas. Furthermore, we found a weaker but significant positive correlation between the TRPD index in the left anterior area and efficacy (r= 0.24, p=0.017), which may suggest that greater efficacy is related to lower cortical activity in the anterior left-brain area (indexed by higher TRPD).

Most of the studies of structural and functional brain changes in burnout included subjects who had severe and long-lasting symptoms and sometimes required at least 50% sick leave for stress-related symptoms for a minimum of 6 months before the study [23]. In the presented study, although it was conducted on a nonclinical burnout sample, the results confirm different brain characteristics in burnout subjects. We observed significantly lower alpha power in the burnout group in the eyes-open condition, which was not reported by previous EEG studies on burnout [29, 49]. This might be associated with the sample characteristics because Luijtelaar et al. [29] tested subjects with more severe burnout symptoms that led to a reduction of their work time of up to 50% for at least 3 months. It seems that the consequences of work-related stress and/or other health problems in their study sample were greater than in our sample of healthy and currently employed full-time workers. Therefore, it seems that burnout severity may be manifested by differences in the EEG power spectrum; however, further comparative analysis conducted among individuals with different burnout levels is required to draw clear conclusions. Referring to Tement et al.'s [49] study, their sample comprised students aged between 19 and 29 with no distinctive burnout outcomes, and their analysis was based on the eyes-closed condition only. Thus, the sample characteristics in all previously presented findings differ significantly, which may result in different study outcomes and lead to inconclusive findings.

## 4. Conclusions

The EEG power spectrum, regulated by anatomically complex homeostatic systems in the various frequency bands, is generally stable in healthy individuals but can be abnormal in some psychiatric disorders due to the dysfunction of this regulation [57]. The presented power analysis showed that in the eyes-open condition the alpha power was lower in the burnout group than in the controls, suggesting that power density might even be sensitive to differences between the healthy and the nonclinical burnout samples.

From the perspective of functional meaning, the reduced alpha power in burnout individuals suggests cortical hyperactivity and may be related to the greater mental effort and possible compensatory mechanisms developed by burnout subjects, as we pointed out in our previous findings [30]. The decreased alpha power is a novel characteristic of burnout syndrome and may indicate different mechanisms compared to depression and fatigue. However, further studies are required to verify these findings in other nonclinical and clinical burnout samples.

Finally, our findings indicate that gender may change the pattern of relations between spectral EEG characteristics and burnout symptoms; therefore, in future studies on burnout, gender should be considered as an important moderating factor.

## Figures and Tables

**Table 1:** Correlation coefficients between alpha power and burnout symptoms in the eyes-open condition (N=95).

Condition	Region	Site	MBI-GS:	MBI-GS:	MBI-GS:
			Exhaustion	Cynicism	Efficacy
Eyes-open	Anterior	Global	-0.2952	-0.2761	0.1425
			p=0.004	p=0.007	p=0.168
		L	-0.2872	-0.2831	0.1380
			p=0.005	p=0.005	p=0.182
		R	-0.2897	-0.2586	0.1424
			p=0.004	p=0.011	p=0.169
	Central	Global	-0.2598	-0.2084	0.1229
			p=0.011	p=0.043	p=0.235
		L	-0.2754	-0.2265	0.1384
			p=0.007	p=0.027	p=0.181
		R	-0.2374	-0.1833	0.1058
			p=0.021	p=0.075	p=0.308
	Posterior	Global	-0.3050	-0.2722	0.1576
			p=0.003	p=0.008	p=0.127
		L	-0.3186	-0.2818	0.1768
			p=0.002	p=0.006	p=0.087
		R	-0.2899	-0.2583	0.1402
			p=0.004	p=0.011	p=0.175

**Table 2:** Correlation coefficients between alpha power and burnout symptoms in the eyes-closed condition (N=95).

Condition	Region	Site	MBI-GS:	MBI-GS:	MBI-GS:
			Exhaustion	Cynicism	Efficacy
Eyes-closed	Anterior	Global	-0.2130	-0.1282	0.1248
			p=0.038	p=0.216	p=0.228
		L	-0.2176	-0.1444	0.1316
			p=0.034	p=0.163	p=0.204
		R	-0.2044	-0.1214	0.1189
			p=0.047	p=0.241	p=0.251
	Central	Global	-0.1933	-0.0931	0.0920
			p=0.061	p=0.370	p=0.375
		L	-0.2184	-0.1212	0.1186
			p=0.033	p=0.242	p=0.252
		R	-0.1627	-0.0620	0.0643
			p=0.115	p=0.551	p=0.536
	Posterior	Global	-0.2139	-0.1336	0.1258
			p=0.037	p=0.197	p=0.224
		L	-0.2250	-0.1390	0.1324
			p=0.028	p=0.179	p=0.201
		R	-0.1967	-0.1151	0.1149
			p=0.056	p=0.267	p=0.267

## Data Availability

The data used to support the findings of this study are available from the corresponding author upon request.
